# 2-Hydroxymelatonin, a Predominant Hydroxylated Melatonin Metabolite in Plants, Shows Antitumor Activity against Human Colorectal Cancer Cells

**DOI:** 10.3390/molecules22030453

**Published:** 2017-03-14

**Authors:** Yi Yang, Rui Zhou, So-Yeon Park, Kyoungwhan Back, Woo Kyun Bae, Kyung Keun Kim, Hangun Kim

**Affiliations:** 1 College of Pharmacy and Research Institute of Life and Pharmaceutical Sciences, Sunchon National University, 255 Jungangno, Sunchon, Jeonnam 57922, Korea; yangyi_520@hotmail.com (Y.Y.); zhourui274@hotmail.com (R.Z.); sinbu17@naver.com (S.-Y.P.); 2 Department of Biotechnology, Bioenergy Research Center, College of Agriculture and Life Sciences, Chonnam National University, 77 Yongbong-ro, Buk-gu, Gwangju 61186, Korea; kback@chonnam.ac.kr; 3 Department of Internal Medicine, Chonnam National University Medical School, 160 Baekseo-ro, Dong-gu, Gwangju 61469, Korea; drwookyun@chonnam.ac.kr; 4 Medical Research Center for Gene Regulation, Brain Korea 21 Project, Chonnam National University Medical School, 160 Baekseo-ro, Dong-gu, Gwangju 61469, Korea; kimkk@chonnam.ac.kr

**Keywords:** melatonin, metabolite, hydroxymelatonin, antitumor, colorectal cancer

## Abstract

2-Hydroxymelatonin is a predominant hydroxylated melatonin metabolite in plants. To investigate whether it has potent cytotoxic effects on colorectal cancer cells, four colorectal cancer cell lines, Caco2, HCT116, DLD1, and CT26, were treated with 2-hydroxymelatonin and melatonin. 2-Hydroxymelatonin had a much lower IC_50_ value than melatonin in the 3-(4,5-dimethylthiazol-2-yl)-2,5-diphenyltetrazolium bromide (MTT) assay. The cytotoxic effect of 2-hydroxymelatonin was much stronger than that of melatonin at high concentrations (1000 or 2000 μM) in HCT116, DLD1, and CT26 cells, but only at intermediate concentrations (250 or 500 μM) in Caco2 cells. The cytotoxicity of 2-hydroxymelatonin was induced through activation of the apoptotic signaling pathway, as confirmed by Hoechst staining and Annexin V-FITC/propidium iodide double labeling of cells treated with a lethal dose (1 mM). However, sub-lethal doses of 2-hydroxymelatonin inhibited the invasive ability of Caco2 cells. Epithelial-mesenchymal transition (EMT) markers were significantly regulated by 2-hydroxymelatonin. Overall, the anti-cancer activity of 2-hydroxymelatonin is more potent than that of melatonin. Taken together, 2-hydroxymelatonin exhibits potent anti-cancer activity against human colorectal cancer cells via induction of apoptosis and inhibition of EMT.

## 1. Introduction

Colorectal cancer is the leading cause of cancer death worldwide. The mortality rate is nearly 33% in developed counties, and even appropriate surgery for patients with local disease has a major effect on survival only at 5 or 10 years [1]. Due to poor progress in the diagnosis and identification of colorectal cancer, screening for specific anti-cancer reagents has become important in colorectal cancer therapy.

Melatonin (*N*-acetyl-5-methoxytryptamine) is a widely studied low molecular weight molecule whose function has been investigated in various types of organisms. In plants, melatonin protects against various types of abiotic stresses [2,3,4] and acts as a growth regulator [5,6,7,8]. Melatonin plays a crucial role in the circadian system to maintain human health [9]. The anticancer activities of melatonin have been reported with several mechanisms elucidated including, modulation of cell cycle and induction of apoptosis [10]; reduction of migration and invasiveness [11]; inhibition of tumor angiogenesis [12]; reactive oxygen species (ROS)-mediated pathways [13]; and immunomodulation [14]. Melatonin in conjunction with chemotherapy has also shown a synergistic effect and reduction of chemotherapy toxicity such as cadiotoxicity [15] and testicular toxicity [16].

Melatonin was recently found to be converted into 2-hydroxymelatonin in plants, and the gene encoding the enzyme that catalyzes melatonin into 2-hydroxymelatonin, melatonin 2-hydroxylase (M2H), was cloned from rice [17]. Some studies indicated that the synthesis rate of 2-hydroxymelatonin is much higher than that of melatonin in rice [18,19,20,21]. Given that many anti-cancer activities of melatonin have been reported, we tested the anti-cancer activity of 2-hydroxymelatonin against colorectal cancer cell lines in this study and further explored the molecular mechanisms underlying the inhibitory effect on invasion.

## 2. Results

### 2.1. 2-Hydroxymelatonin Has Potent Cytotoxic Effects on Human Colorectal Cancer Cells

**Figure 1:**
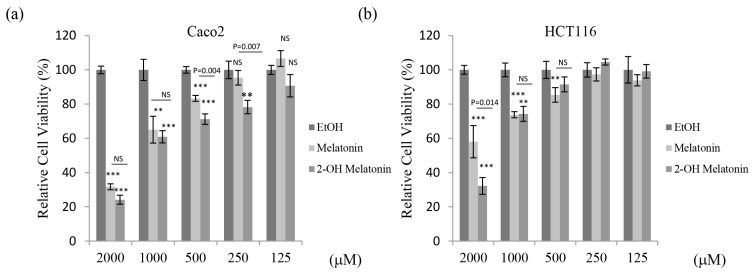

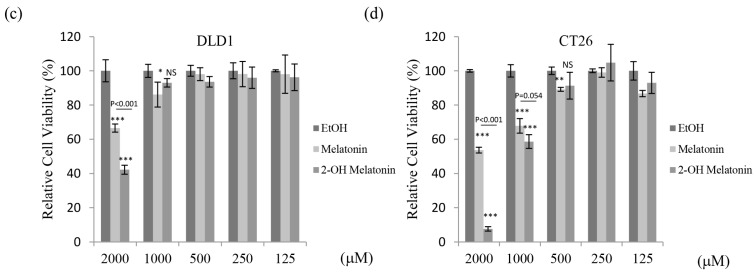
Cytotoxic effects of 2-hydroxymelatonin on human colorectal cancer cells. (**a**–**d**) Percentage viability of Caco2 (**a**), HCT116 (**b**), DLD1 (**c**), and CT26 (**d**) cells treated with melatonin or 2-hydroxymelatonin. Cells were treated with 125–2000 µM melatonin or 2-hydroxymelatonin dissolved in ethanol for 48 h, and cell viability was measured by the MTT assay. Data represent means ± standard error of the mean, *n* = 3. * *p* < 0.05; ** *p* < 0.01; *** *p* < 0.001; NS, no significant difference compared with the ethanol-treated group. EtOH, ethanol; 2-OH Melatonin, 2-hydroxymelatonin.

To investigate whether 2-hydroxymelatonin has stronger cytotoxic effects than melatonin, the 3-(4,5-dimethylthiazol-2-yl)-2,5-diphenyltetrazolium bromide (MTT) assay was performed using both compounds. 2-Hydroxymelatonin had a much stronger cytotoxic effect than melatonin on HCT116, DLD1, and CT26 cells at high concentrations (1000 or 2000 μM) (Figure 1b–d). In particular, 2-hydroxymelatonin reduced CT26 cell viability by 90%. By contrast, 2-hydroxymelatonin had a much stronger cytotoxic effect than melatonin on Caco2 cells at intermediate concentrations (250 or 500 μM) (Figure 1a), and both compounds reduced Caco2 cell viability by ~70% at a concentration of 2000 μM. However, 2-hydroxymelatonin had a significantly lower IC_50_ value than melatonin in all four of these colorectal cancer cell lines (Table 1). Together, these results suggest that 2-hydroxymelatonin has potent cytotoxic effects on colorectal cancer cell lines.

### 2.2. A Lethal Concentration of 2-Hydroxymelatonin Induces Apoptosis in Caco2 Cells

**Figure 2:**
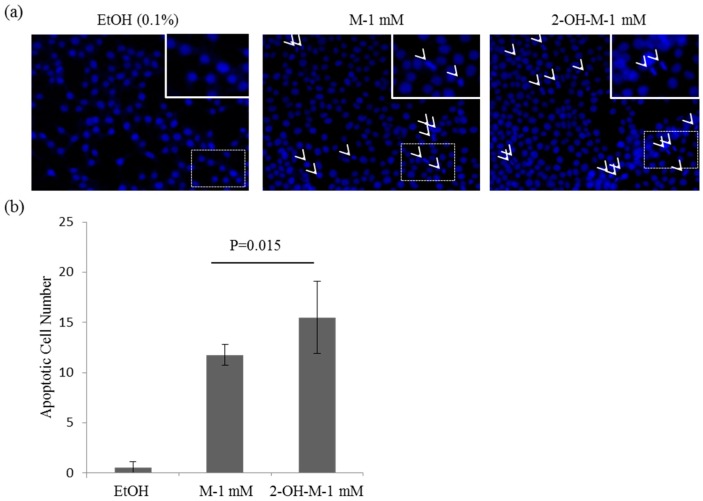
Induction of nuclear condensation in Caco2 cells by melatonin or 2-hydroxymelatonin. (**a**) Hoechst 33258 staining of Caco2 cells treated with melatonin or 2-hydroxymelatonin dissolved in ethanol. Arrows indicate cells with condensed or fragmented nuclei. Representative images from three independent experiments are shown. Enlarged images are shown in the top right inset of the image; (**b**) Quantification of condensed or fragmented nuclei in cells treated with melatonin or 2-hydroxymelatonin. Data represent mean ± standard error of the mean, *n* = 3. EtOH, ethanol; M, melatonin; 2-OH-M, 2-hydroxymelatonin.

To determine whether the cytotoxicity of 2-hydroxymelatonin is due to the induction of apoptosis, Caco2 cells treated with a lethal concentration of 2-hydroxymelatonin (1000 μM) or melatonin (1000 μM) were stained with Hoechst 33258 and their nuclear morphology was observed. Condensed nuclei were seen in Caco2 cells treated with 2-hydroxymelatonin or melatonin (Figure 2a). The numbers of cells with condensed nuclei are quantified in Figure 2b. There were significantly more cells with condensed nuclei in 2-hydroxymelatonin-treated samples than in melatonin-treated samples. These results suggest that 2-hydroxymelatonin induces apoptosis more strongly than melatonin at the same lethal concentration (1000 μΜ).

**Figure 3:**
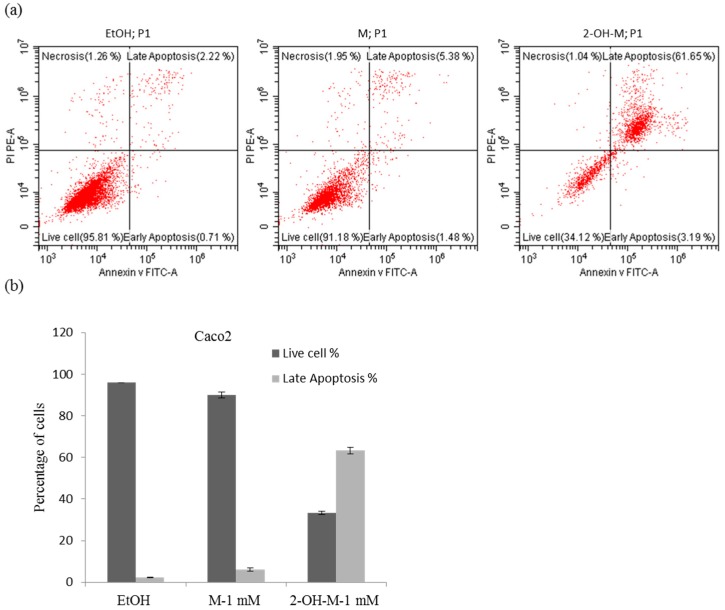
Induction of apoptosis in Caco2 cells by 2-hydroxymelatonin, as determined by staining with Annexin V-FITC and propidium iodide (PI). (**a**) Annexin V-FITC and PI staining of Caco2 cells treated with 1 mM melatonin or 2-hydroxymelatonin; (**b**) Quantification of apoptosis induction in cells treated with 1 mM melatonin or 2-hydroxymelatonin. The results are representative of three experiments. EtOH, ethanol; M, melatonin; 2-OH-M, 2-hydroxymelatonin.

To further determine the percentage of apoptotic cells, flow cytometric analysis of cells stained with Annexin V-FITC and propidium iodide (PI) was performed. An extremely large population of 2-hydroxymelatonin-treated Caco2 cells was both Annexin V-FITC- and PI-positive (Figure 3a). Quantitative data revealed that there were significantly more apoptotic Caco2 cells in 2-hydroxymelatonin-treated samples than in melatonin-treated samples at a concentration of 1000 μM (Figure 3b). Together, these findings show that the cytotoxicity of 2-hydroxymelatonin is due to activation of the apoptotic signaling pathway.

### 2.3. Sub-Lethal Concentrations of 2-Hydroxymelatonin Inhibit the Invasive Ability of Caco2 Cells

**Figure 4:**
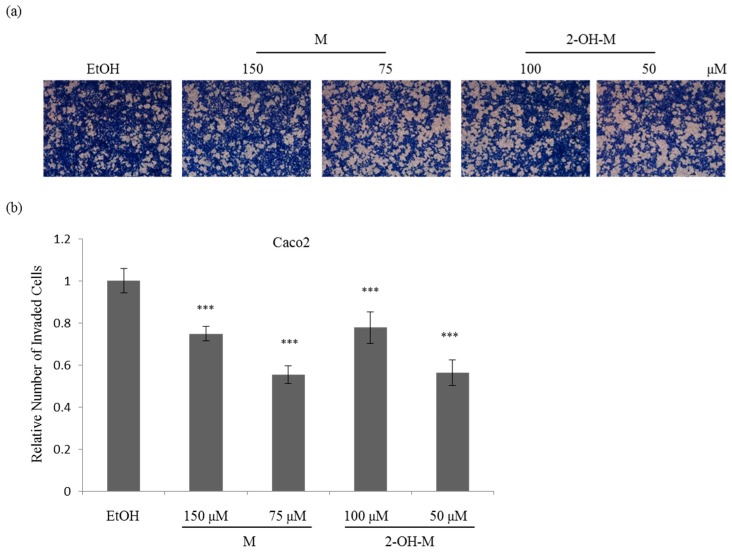
Inhibition of the invasive ability of Caco2 cells by treatment with sub-lethal concentrations of melatonin or 2-hydroxymelatonin. (**a**–**b**) Invasion assay of Caco2 cells treated with melatonin or 2-hydroxymelatonin dissolved in ethanol (**a**), and quantification of the number of invaded cells in each group (**b**). Representative images from three independent experiments are shown. Data represent mean ± standard error of the mean, *n* = 3. *** *p* < 0.001 compared with the ethanol-treated group. EtOH, ethanol; M, melatonin; 2-OH-M, 2-hydroxymelatonin.

To further explore the anti-cancer activity of 2-hydroxymelatonin and melatonin, cell invasion was examined following treatment with one-tenth or one-twentieth of the IC_50_ concentrations of both compounds, which did not have cytotoxic effects (sub-lethal concentrations). There were fewer invaded cells in samples treated with melatonin or 2-hydroxymelatonin than in the ethanol-treated control group (Figure 4a). Quantitative data showed that 2-hydroxymelatonin and melatonin inhibited Caco2 cell invasion to a similar extent (Figure 4b). Interestingly, Caco2 cell invasion was inhibited 25% more by treatment with 2-hydroxymelatonin or melatonin at one-twentieth of the IC_50_ concentration than at one-tenth of the IC_50_ concentration. All these results indicate that sub-lethal concentrations of 2-hydroxymelatonin inhibit Caco2 cell invasion.

### 2.4. 2-Hydroxymelatonin Inhibits Caco2 Cell Invasion through Epithelial-Mesenchymal Transition (EMT) Regulation

**Figure 5:**
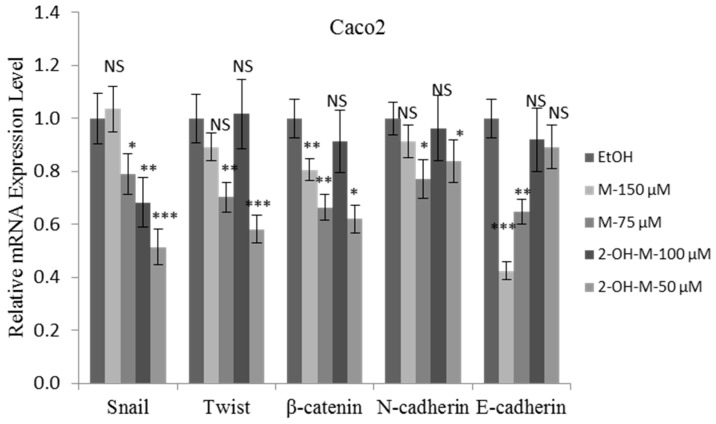
mRNA expression of Epithelial–mesenchymal transition (EMT) markers upon treatment with sub-lethal concentrations of melatonin or 2-hydroxymelatonin. Quantitative analysis of the mRNA levels of EMT markers in Caco2 cells treated with melatonin or 2-hydroxymelatonin dissolved in ethanol for 24 h. Data represent mean ± standard error of the mean, *n* = 3. * *p* < 0.05; ** *p* < 0.01; *** *p* < 0.001; NS, no significant difference compared with the ethanol-treated group. EtOH, ethanol; M, melatonin; 2-OH-M, 2-hydroxymelatonin.

We tested whether EMT is involved in the inhibition of Caco2 cell invasion by 2-hydroxymelatonin. The mRNA levels of Snail, Twist, and *N*-cadherin were significantly downregulated in Caco2 cells treated with 2-hydroxymelatonin at one-twentieth (50 μΜ) of the IC_50_ concentration for 24 h (Figure 5), which was consistent with the previous invasion results. All the results suggest that 2-hydroxymelatonin inhibits Caco2 cell invasion by suppressing the EMT pathway. However, 2-hydroxymelatonin did not significantly affect the mRNA level of E-cadherin. In addition, 2-hydroxymelatonin decreased the mRNA levels of β-catenin (Figure 5), which indicates that the Wnt pathway is involved in this inhibition.

## 3. Discussion

Melatonin, a well-known hormone found in various types of organisms, has various anti-cancer activities. 2-Hydroxymelatonin is metabolized from melatonin by melatonin 2-hydroxylase (M2H) in plants. The biological activities of 2-hydroxymelatonin remain to be examined [17]; therefore, we tested the anti-cancer activity of 2-hydroxymelatonin against colorectal cell lines. In this study, we demonstrated that 2-hydroxymelatonin had stronger cytotoxic effects than melatonin on colorectal cancer cell lines. This is the first anti-cancer activity reported for 2-hydroxymelatonin. Moreover, one-tenth and one-twentieth of IC_50_ concentrations of 2-hydroxymelatonin and melatonin inhibited Caco2 cell invasion.

EMT is an essential step in cell metastasis, and many EMT-inducing transcription factors, such as Snail and Twist1, are related to tumor invasion and metastasis. Snail, a transcription factor, directly represses E-cadherin transcription [22,23]. Twist also plays an essential role in cell migration and invasion. Suppression of Twist specifically inhibits the ability of cells to metastasize from the mammary gland to the lung [24]. The mRNA levels of Snail and Twist were significantly downregulated by 2-hydroxymelatonin or melatonin treatment at one-twentieth of the IC_50_ concentration, which suggests that 2-hydroxymelatonin inhibits Caco2 cell invasion by modulating the EMT pathway.

β-Catenin plays a signaling role as a component of the Wnt pathway. β-catenin accumulates and specific target genes are transcriptionally activated upon Wnt signaling activation [25]. β-catenin transcriptional activity is required in newly formed mesenchymal-type cardiac cushions during EMT in both zebrafish [26,27] and mouse [28] embryos. Therefore, there is a close relationship between the Wnt signaling pathway and the EMT process. In this study, treatment with sub-lethal concentrations of 2-hydroxymelatonin significantly downregulated β-catenin in Caco2 cells, which indicates that the Wnt signaling pathway is involved in the inhibition of Caco2 cell invasion.

All these results imply that 2-hydroxymelatonin is a potential anti-cancer reagent and can be used to induce cytotoxicity or inhibit metastasis in further research.

## 4. Materials and Method

### 4.1. Cell Proliferation Assay

The proliferation and viability of cells were measured using the MTT colorimetric assay (Sigma-Aldrich, St. Louis, MO, USA). Cells were seeded at a density of 2.5 × 10^3^ cells/well in 96-well plates and grown for 48 h in the presence of 2-hydroxymelatonin (Toronto Research Chemicals, ON, Canada) or melatonin (Sigma-Aldrich). MTT was added for 4 h. DMSO was added after removing the medium. Absorbance at 540 nm was determined using a microplate reader with Gen 5 (2.03.1) software (BioTek, VT, USA)

### 4.2. Cell Culture

Human (Caco2, HCT116, and DLD1) and mouse (CT26) colorectal cancer cells were cultured in DMEM supplemented with 10% fetal bovine serum and 1% penicillin–streptomycin solution in a humidified 5% CO_2_ atmosphere at 37 °C.

### 4.3. Hoechst Staining

Cells were cultured as described above. After treatment with 2-hydroxymelatonin or melatonin for 24 h, cells were washed thrice with phosphate-buffered saline (PBS), fixed in 4% paraformaldehyde for 15 min, and then incubated in 0.1% Triton X-100 (Sigma-Aldrich) for 30 min. Subsequently, the samples were stained with Hoechst 33257 (Sigma-Aldrich) at room temperature for 30 min. The cells were mounted onto a glass slide after being washed twice with PBS. The slides were analyzed using a Nikon Eclipse 400 fluorescence microscope (Nikon Instech Co., Ltd., Kawasaki, Japan), and the number of fragmented nuclei per captured image was evaluated.

### 4.4. Flow Cytometric Analysis

Caco2 cells were plated in 6-well plates at a density of 2 × 10^5^ cells/well, cultured overnight, treated with 1 mM melatonin or 2-hydroxymelatonin for 24 h, trypsinized, and washed with ice-cold PBS. All cells were re-suspended in 100 μL of 1× Binding Buffer containing 5 μL of a 50 μg/mL stock PI solution (BD Biosciences, San Jose, CA, USA) and 1 μL of Annexin V-FITC (BD Biosciences) for 15 min in the dark, and then analyzed by flow cytometry using a CytoFLEX instrument (Beckman Coulter Life Sciences, Indianapolis, IN, USA).

### 4.5. Cell Invasion Assay

Invasion assays were performed in Boyden chambers (Corning, New York, NY, USA). Transwell chambers pre-coated with 1% gelatin were used, as previously described [29]. Cells (2 × 10^6^) in 120 μL of medium (DMEM containing 0.2% bovine serum albumin dissolved in PBS) containing or lacking various concentrations of melatonin or 2-hydroxymelatonin were seeded in the upper chamber. Then, 400 μL of medium containing 10 μg/mL fibronectin was added to the lower chamber to serve as a chemotactic agent. After incubation for 24 h, cells in the upper chamber were fixed with a Diff-Quik kit (Sysmex, Kobe, Japan). Cells attached to the underside of the transwell were stained and counted underneath an upright microscope (five fields/chamber). Each invasion assay was repeated in three independent experiments.

### 4.6. Western Blotting

Treated cells were washed twice with ice-cold PBS and lysed in lysis buffer. Anti-β-catenin and anti-α-tubulin antibodies were purchased from Cell Signaling Technology (Danvers, MA, USA) and anti-E-cadherin and anti-N-cadherin antibodies were purchased from BD Biosciences. These antibodies were detected with a horseradish peroxidase-conjugated secondary antibody (Thermo Fisher Scientific, Waltham, MA, USA) using an Immobilon Western Chemiluminescent HRP Substrate Kit (Merck Millipore, Darmstadt, Germany) and luminescence imaging (Image Quant LAS 4000 mini). Bands were measured using Multi-Gauge 3.0, and their relative density was calculated based on the density of the α-tubulin band in each sample. 

### 4.7. Quantitative Real-Time PCR

Quantitative real-time PCR was performed as described previously [30]. Briefly, total RNA was isolated from human lung cancer cells using RNAiso Plus (TaKaRa, Otsu, Shiga 520-2193, Japan) according to the manufacturer’s instructions. Total RNA (1 µg) from each group of treated cells was converted to cDNA using M-MLV reverse transcriptase (Invitrogen, Carlsbad, CA, USA) and SYBR Green (Enzynomics, Seoul, Korea). The primers used for real-time PCR were as follows: E-cadherin (forward) 5′-CAGAAAGTTTTCCACCAAAG-3′ and (reverse) 5′-AAATGTGAGCAATTCTGCTT-3′; Snail (forward) 5′-TCCCGGGCAATTTAACAATG-3′ and (reverse) 5′-TGGGAGACACATCGGTCAGA-3′; N-cadherin (forward) 5′-CTCCTATGAGTGGAACAGGAACG-3′ and (reverse) 5'-TTGGATCAATGTCATAATCAAGTGCTGTA-3′; Twist (forward) 5′-CGGGAGTCCGCAGTCTTA-3′ and (reverse) 5′-TGAATCTTGCTCAGCTTGTC-3′; β-catenin (forward) 5′-AAAATGGCAGTGCGTTTAG-3′ and (reverse) 5′-TTTGAAGGCAGTCTGTCGTA-3′; and GAPDH (forward) 5′-ATCACCATCTTCCAGGAGCGA-3′ and (reverse) 5’-AGTTGTCATGGATGACCTTGGC-3′. Real-time PCR and analysis were performed using CFX (Bio-Rad, Hercules, CA, USA).

### 4.8. Statistical Analysis

All experiments were assayed in triplicates (*n* = 3). Results are reported as mean ± standard error of the mean (SEM). All statistical analyses were performed using the SPSS version 17. Treatment effects were determined using one-way ANOVA post-hoc analysis. The level of significance was set at *p* < 0.05.

## 5. Conclusions

In summary, 2-hydroxymelatonin exhibits potent anti-cancer activity against human colorectal cancer cells. At a lethal concentration, 2-hydroxymelatonin causes cytotoxicity by inducing apoptosis. At a sub-lethal concentration, 2-hydroxymelatonin inhibits the invasive potential of cells and decreases the expression of EMT markers.

## Figures and Tables

**Table 1:** IC_50_ values of melatonin and 2-hydroxymelatonin.

Viability (%)	Caco2		HCT116		DLD1		CT26	
	M	2-OH-M	M	2-OH-M	M	2-OH-M	M	2-OH-M
2000 μM	31.7	24.1	58.0	32.2	66.5	42.2	53.7	7.6
1000 μM	65.1	60.9	73.8	74.3	86.1	93.0	67.8	58.6
500 μM	83.3	71.2	85.4	91.5	98.0	93.6	89.1	91.3
250 μM	95.3	78.3	97.4	104.6	98.1	96.0	99.1	104.8
IC_50_ (μM)	1324.6 ± 69.18	1010.3 ± 28.0	2537.4 ± 311.4	1482.3 ± 67.4	3617.1 ± 594.3	2136.5 ± 84.8	1980.9 ± 14.8	1036.1 ± 25.7
*p*-value	0.014		0.029		0.043		<0.001	

Data represent the mean value from three independent experiments. *p*-value was obtained between the M and 2-OH-M treated group of each cell. M: melatonin; 2-OH-M: 2-hydroxymelatonin.
